# Age at Diagnosis for Lung, Colon, Breast, and Prostate Cancers: An International Comparative Study

**DOI:** 10.1200/GO.22.47000

**Published:** 2022-05-05

**Authors:** Hana Zahed, Xiaoshuang Feng, Mahdi Sheikh, Melina Arnold, Freddie Bray, Jacques Ferlay, Meredith Shiels, Hilary Robbins

**Affiliations:** ^1^ International Agency for Research on Cancer (IARC/WHO), Lyon, France; ^2^ National Cancer Institute, Rockville, MD

## Abstract

Differences in the age at diagnosis for lung, colon, breast, and prostate cancers have been reported between low- and middle-income countries (LMICs) and high-income countries (HICs). However, this may be influenced by differences in the population age distributions across countries. We aimed to compare the median age at diagnosis for these cancers after adjusting for population age differences.

**METHODS:**

We analyzed data from the Cancer Incidence in 5 Continents (CI5) Volume XI database. It includes information on cancer diagnoses during 2008 to 2012 from cancer registries in 66 countries. We calculated crude median ages at diagnosis for each cancer in each country, and then performed indirect standardization using the age-specific UN world population estimate to remove the influence of population age structure.

**RESULTS:**

Overall, the adjustment for population age structure tended to increase the median ages at diagnosis in LMICs which have younger populations, and decrease them in HICs which have older populations. After standardization, differences between the youngest and oldest median ages of diagnosis across cancer sites were: 11 years for lung cancer (youngest median age observed was 61 in Bulgaria *v* 71 in Bahrain), 10 years for colon cancer (59 in Iran *v* 69 New Zealand), 10 years for breast (49 in Algeria *v* 59 Iceland), and 8 years for prostate cancer (65 in USA *v* 73 in the Philippines). LMICs had younger ages at diagnosis for colon cancer but older ages at diagnosis for prostate cancer as compared with HICs. Countries with higher smoking prevalence had younger ages at lung cancer diagnosis (*P* value Pearson correlation = 0.0025).

**CONCLUSION:**

For lung, colon, breast, and prostate cancers, the differences across countries in the median age at diagnosis range from 8 to 11 years after adjusting for population age distribution. These differences likely reflect population-level variation in risk factors and screening.

